# Debate: Social media in children and young people – time for a ban? From polarised debate to precautionary action – a population mental health perspective on social media and youth well‐being

**DOI:** 10.1111/camh.70033

**Published:** 2025-09-10

**Authors:** Oonagh Welsey‐Smith, Terry Fleming

**Affiliations:** ^1^ Te Puna Hauora/School of Health Te Herenga Waka/Victoria University of Wellington Wellington New Zealand

**Keywords:** Adolescence, mental health, policy, social media

## Abstract

Adolescents spend much of their daily lives online, with social media a central part of their digital environment. While findings are complex, evidence increasingly points to small but relatively consistent harms, particularly for those meeting criteria for problematic use. At the population level, these effects are concerning, given the extraordinary prevalence of exposure to social media, rising rates of problematic use and adolescents' vulnerability to mental ill‐health. The complexity of determining causality should not delay action: from a public health perspective, precautionary measures are warranted. We argue for a comprehensive approach that includes effective age verification and age‐appropriate protection, regulation of harmful design features for all ages and inclusion of young people's voices. Just as offline environments include regulation and guidance, digital environments must be shaped to support individual and community needs.

Fifteen‐year‐olds worldwide spend an average of 7 hours a day on digital devices, with a third of 11‐ to 15‐year‐olds continuously connected to social media (OECD, 2025). The digital environment is central to young people's lives, making it vital to examine its impact and to ensure that the online protections are provided. While causality between social media use and harms remains under investigation, the repeated reports of harmful associations justify a precautionary population health response—one that includes but is not limited to regulatory action.

A wide range of methods has been used to study the relationship between social media and adolescent health. Cross‐sectional and longitudinal studies have found small positive associations between some indicators of social media use and mental ill‐health; however, there are benefits such as fostering social connection for some. (OECD, 2025). These results contrast with time‐series analyses that report sharp increases in adolescent distress linked to rising social media use at a population level (Mojtabai, 2024; Twenge, Joiner, Rogers, & Martin, 2017). Alternative explanations such as parenting changes, mental health literacy or climate concerns struggle to fully account for the rapidity and timing of these trends. The divergence in findings has fuelled a polarised debate.

Against this backdrop, nuanced analyses are essential. Recent research highlights that the greatest risks lie not in light or moderate use, but in *problematic social media use* (PSMU)– maladaptive use beyond an individual's control, which interferes with daily functioning, and in *excessive time online*. Across cross‐sectional, longitudinal, ecological and meta‐analytical studies, PSMU and heavy use have been linked with adverse outcomes, including higher odds of distress and anxiety, inadequate sleep, disordered eating, school avoidance and unfavourable cardiometabolic markers (Boniel‐Nissim et al., 2022; Liu et al., 2022; Mojtabai, 2024; Nagata, Lee, Hur, & Baker, 2025). While associations are generally small to moderate, they have been repeatedly demonstrated in large, high‐quality datasets (Boniel‐Nissim et al., 2022; Nagata et al., 2025; Nagata, Lee, et al., 2025). Further, and critically, PSMU has increased 50% worldwide over four years (Boniel‐Nissim et al., 2024).

There is growing consensus that beneficial, neutral and harmful effects coexist, and that behind the aggregate statistics lie heterogeneous complex relationships. Susceptibility to harm is shaped by multiple factors including individual characteristics, developmental stage, online experiences and social contexts – particularly family and peer relationships (Nagata, Otmar, et al., 2025; Orben, Przybylski, Blakemore, & Kievit, 2022). Platform functionalities and affordances (e.g. algorithms, recommender systems, personalised advertising and privacy limitations) contribute to risks. Current research is focussed on distinguishing the influence of problematic from intensive use, and on clarifying the extent to which a bidirectional relationship exists – in which each factor mutually reinforces the other, creating a self‐fulfilling cycle. While the literature is evolving rapidly, definitive conclusions may remain elusive. This is common in population health, which is shaped by multiple interacting factors and shifting contexts.

At the population level, even small effects translate into substantial consequences when many people are exposed to them, particularly when the baseline risk is already high – as it is for mental ill‐health in this age group (Rutledge & Loh, 2004). Additionally, social media impacts compound across other domains, for example, educational engagement and physical health (Boniel‐Nissim et al., 2022; Nagata, Lee, et al., 2025). Moreover, population‐wide exposure matters: even those who avoid social media live in a society where it shapes norms—such as attitudes towards body size, life expectations and cultural values.

From a population health perspective, taking action to prevent harm is often necessary even while evidence is evolving. The research provides justification for a precautionary approach. The rapid rise in PSMU underscores this urgency. (Boniel‐Nissim et al., 2024).

As with other public health challenges, a multifaceted approach is required. This should include individual‐level interventions such as education responses and parental guidance, as well as community‐level actions such as moderating device use in schools and providing ample offline opportunities (OECD, 2025). Crucially, an effective response must also include the safety of online environments and policy settings governing these.

There are a number of regulatory responses being enacted worldwide, ranging from comprehensive approaches such as the EU Digital Services Act (DSA), to narrower responses such as Australia's social media ban. While some form of age restriction is important, these overlook the reality that individuals of all ages are vulnerable to misinformation, scams and problematic use. Blanket bans also fail to acknowledge benefits from social media and harms from illicit workarounds. More comprehensive approaches such as the EU's DSA, adopt a ‘safety by design’ framework, requiring that all digital service providers assess and mitigate platform‐related harms. Such approaches incorporate online age verification, default privacy and data settings, and requirements for effective content moderation (OECD, 2025).

From a population mental health perspective, effective regulatory action should:

1. *Establish a duty of care for digital platforms*. Regulatory frameworks must recognise that the responsibility for protecting users of digital products lies with the providers of digital services. Clearly defined duties of care should be established. These must include the ability to monitor and enforce standards.
2. *Address both harmful content and problematic platform design features*. Regulation must address harmful content and design features such as default use of AI, algorithms, targeted advertising and autoplay.
3. *Adopt online age verification tools*. These are required to address social media risks and for regulating other concerns such as online gambling and alcohol sales. Although workarounds for these measures will exist, challenges in enforcement of regulations require careful attention – not dismissal of the approach.
4. *Recognise developmental needs*. Adolescence is a period of heightened susceptibility to mental ill‐health and long‐term habit formation. Gender‐specific windows of susceptibility to social media related harm (ages 11–13 for girls and 14–15 for boys) underscore the need for age‐appropriate protections (Orben et al., 2022). Developmentally informed age restrictions have long been utilised in areas such as alcohol and driving restrictions.
5. *Consider targeted rather than blanket responses*. Policy‐makers should explore the value of targeted compared to general restrictions. For example, applying age restrictions only on certain platforms or the use of approaches such as banning algorithms/targeted advertisements/ autoplay for accounts belonging to young people.
6. *Include young people's voices and equity analyses*. Policies must balance safeguarding with respect for youth rights and perspectives. Their lived experience is vital to crafting responses that are effective and legitimate. Any response can reduce or widen inequities and harms for Indigenous and minoritised groups. Equity analyses and actions must be central.

The evidence to date suggests that mental health has rapidly declined among youth and that social media use has complex and often harmful effects. The largely unregulated, self‐governing nature of platforms is out of keeping with the importance of digital environments in young people's lives. Online spaces are real‐world environments and require tangible safeguards. At the same time, young people's rights and the mental health of all age groups are critical. Multilevel nuanced actions are needed to achieve real gains.

## Conflict of interest statement

The author has no conflicts of interest to disclose.

## Funding information

No funders declared.

## Ethics statement

No ethics approval was required for this debate article.

## Data Availability

Data sharing is not applicable to this article as no new data were created or analysed.

## References

[camh70033-bib-0001] Boniel‐Nissim, M. , Marino, C. , Galeotti, T. , Blinka, L. , Ozoliņa, K. , Craig, W. , … & van den Eijnden, R. (2024). Report: A focus on adolescent social media use and gaming in Europe, Central Asia and Canada: Health Behaviour in School‐aged Children international report from the 2021/2022 survey. World Health Organization. Regional Office for Europe. https://iris.who.int/handle/10665/378982

[camh70033-bib-0002] Boniel‐Nissim, M. , van den Eijnden, R. , Furstova, J. , Marino, C. , Lahti, H. , Inchley, J. , … & Badura, P. (2022). International perspectives on social media use among adolescents: Implications for mental and social well‐being and substance use. Computers in Human Behavior, 129, 107144.

[camh70033-bib-0003] Liu, M. , Kamper‐DeMarco, K.E. , Zhang, J. , Xiao, J. , Dong, D. , & Xue, P. (2022). Time spent on social media and risk of depression in adolescents: A dose‐response meta‐analysis. International Journal of Environmental Research and Public Health, 19, 5164. 35564559 10.3390/ijerph19095164 PMC9103874

[camh70033-bib-0004] Mojtabai, R. (2024). Problematic social media use and psychological symptoms in adolescents. Social Psychiatry and Psychiatric Epidemiology, 59, 2271–2278. 38584201 10.1007/s00127-024-02657-7 PMC11522145

[camh70033-bib-0005] Nagata, J.M. , Lee, C.M. , Hur, J.O. , & Baker, F.C. (2025). What we know about screen time and social media in early adolescence: A review of findings from the adolescent brain cognitive development study. Current Opinion in Pediatrics, 37, 357–364. 40172268 10.1097/MOP.0000000000001462 PMC12259366

[camh70033-bib-0006] Nagata, J.M. , Otmar, C.D. , Shim, J. , Balasubramanian, P. , Cheng, C.M. , Li, E.J. , … & Baker, F.C. (2025). Social media use and depressive symptoms during early adolescence. JAMA Network Open, 8, e2511704. 40397441 10.1001/jamanetworkopen.2025.11704 PMC12096259

[camh70033-bib-0007] OECD . (2025). Report: How's life for children in the digital age? Paris: OECD Publishing. 10.1787/0854b900-en

[camh70033-bib-0008] Orben, A. , Przybylski, A.K. , Blakemore, S.‐J. , & Kievit, R.A. (2022). Windows of developmental sensitivity to social media. Nature Communications, 13, 1649. 10.1038/s41467-022-29296-3 PMC8960761 35347142

[camh70033-bib-0009] Rutledge, T. , & Loh, C. (2004). Effect sizes and statistical testing in the determination of clinical significance in behavioral medicine research. Annals of Behavioral Medicine, 27, 138–145. 15026298 10.1207/s15324796abm2702_9

[camh70033-bib-0010] Twenge, J. , Joiner, T. , Rogers, M. , & Martin, G. (2017). Increases in depressive symptoms, suicide‐related outcomes, and suicide rates among U.S. adolescents after 2010 and links to increased new media screen time. Clinical Psychological Science, 6, 3–17.

